# Factors associated with willingness to participate in free HIV test among general residents in Heilongjiang, Northeast China

**DOI:** 10.1186/1471-2334-12-256

**Published:** 2012-10-12

**Authors:** Lili Yuan, Xin Li, Xiaoxia Li, Jingli Shi, Liying Jiang, Chundi Zhang, Xiujing Yang, Yeli Zhang, Yashuang Zhao

**Affiliations:** 1 Harbin Medical University, Harbin, Heilongjiang Province, Heilongjiang, China; 2 Department of Epidemiology, Public Health College, Harbin Medical University, Harbin, Heilongjiang Province, China; 3 Department of Public Health, Mudanjiang Medical College, Mudanjiang, Heilongjiang Province, China; 4 Qiqihaer Center for Disease Control and Prevention, Qiqihaer, Heilongjiang Province, China; 5 Qiqihaer Medical College, Qiqihaer, Heilongjiang Province, China; 6 Clinical Laboratory, Third Affiliated Clinical Hospital of Qiqihaer Medical College, Qiqihaer, Heilongjiang Province, China; 7 Disease Control Office, Health Department of Heilongjiang Province, Heilongjiang Province, China

**Keywords:** HIV, Knowledge, Public stigma, Willingness, General individuals

## Abstract

**Background:**

The human immunodeficiency virus (HIV) is spreading from high-risk groups, such as men who have sex with men (MSM) and sex workers, to the general population in China. This study examined the willingness of general residents in Heilongjiang, Northeast China, to participate in free HIV testing in the nearest health care setting, and the factors that may affect participation, including demographic characteristics, HIV-related knowledge, and stigma.

**Methods:**

A cross-sectional study was conducted in Heilongjiang Province. All residents aged 15–69 years in two communities in urban areas (September 2007) and four villages in rural areas (April 2008) were recruited using stratified cluster sampling. A total of 4050 residents were interviewed using an anonymous questionnaire. Univariate and multivariate log-binomial regression were used to analyze factors affecting willingness to undergo HIV testing.

**Results:**

The proportions of participants who were willing to participate in free HIV testing was 73.0% in urban residents and 78.8% in rural residents. Multivariate regression analysis among urban participants showed that greater knowledge of HIV transmission misconceptions (relative risk (RR) = 1.02, 95% confidence interval (CI): 1.00–1.04, *P* = 0.021) and the awareness that an apparently healthy person can be an HIV carrier (RR = 1.12, 95%CI: 1.03–1.21, *P* = 0.007) was significantly associated with greater willingness to participate in free HIV testing. Among rural participants, greater knowledge of HIV transmission modes (RR = 1.03, 95%CI: 1.01–1.06 *P* = 0.001) and the awareness that an apparently healthy person can be an HIV carrier (RR = 1.07; 95%CI: 1.01–1.13 *P* = 0.019) was significantly associated with greater willingness to participate.

**Conclusions:**

The overall level of willingness to accept free HIV testing is high, and is higher in rural residents than in urban residents in Heilongjiang. knowledge of HIV transmission misconceptions and that an apparently healthy person can be a carrier for HIV were associated with willingness to accept free HIV testing among urban residents, while knowledge of HIV transmission modes and that an apparently healthy person can be a carrier for HIV were associated with willingness to accept free HIV testing among rural residents.

## Background

Human immunodeficiency virus (HIV) reached all 31 provinces in China by 1998; the first indigenous Chinese acquired immunodeficiency syndrome (AIDS) case was detected in 1989 [1]. An assessment by the Chinese Ministry of Health, the Joint United Nations Program on HIV/AIDS and the World Health Organization suggested that approximately 740,000 were HIV infections in China at the end of 2009 [2], while the cumulative number of HIV infections actually diagnosed by the end of 2009 was 326,000 in China [2]. It was reported that 44.1% of HIV infections were not aware of their infection [2]. There were 13715 newly-diagnosed HIV infections in 2009 in China, 55.7% of which occurred through sexual transmission, and it was estimated that there were actually 48000 new HIV infections in China in 2009, 74.7% occurring through sexual transmission [2]. Heilongjiang Province, in northeast China, is still an area of low HIV/AIDS incidence and prevalence, but HIV infection has been rapidly spreading to the general population [3]. The incidence rate increased from 0.16 per 100,000 in 2004 to 0.33 per 100,000 in 2006, to 0.91 per 100,000 in 2009 in Heilongjiang Province. 66.5% people who were newly diagnosed and reported in 2009 acquired their infection through sexual transmission in Heilongjiang Province.

Testing for HIV is an important component of an effective AIDS prevention and control campaign [4,5]. Studies indicated that HIV-infected persons reduced high-risk behaviour substantially when they became aware of their infection [6,7]. However, a major barrier to effective prevention and control efforts in China is that most infected people are not aware of their serostatus [1,8]. Although willingness to undergo testing and taking part in testing are two different dimensions along a continuum of HIV prevention, willingness to be tested for HIV before taking concrete action is an important step in the HIV prevention process [9]. In China, studies about willingness to take HIV tests focused primarily on high-risk groups such as sex workers [10], rural-to-urban migrants [11], and commercial blood donors [12]. There have been few studies in the general population about willingness to take HIV tests. One study in Guizhou Province, China, investigated the acceptance of voluntary counselling and testing (VCT) in VCT centre among adults (18–45 years) [5]. However, our study focused on the willingness to undergo testing if free HIV tests were provided at the nearest health care facility, which offered greater accessibility and convenience than a free HIV test in a distant VCT centre.

This study, in Heilongjiang Province, examined (a) the level of willingness of residents to participate in free HIV testing; (b) differences in the willingness between urban and rural residents; (c) the effect of demographic characteristics, HIV-related knowledge and stigma on willingness; and (d) the main reasons for participants’ disinclination to be tested. The outcome of the study should help researchers to determine the feasibility of instituting HIV testing, and to identify factors and reasons for participants’ disinclination to be tested.

## Methods

### Participants and procedures

The study population, aged 15–69 years, was drawn from rural (September 2007) and urban areas (April 2008) using stratified sampling methods. First, two cities, Qiqihaer and Mudanjiang representing a middle socioeconomic level in urban areas (The population density is relatively high, usually more than 100,000 people; people mainly engage in non-agricultural industries), were selected from a total of seven cities. Then two communities of a middle socioeconomic level were selected respectively in the two cities, and all residents of selected communities were invited to participate. Fuyu and Dongning counties with a middle socioeconomic level in rural areas (The population density is relatively scarce; people mainly engage in agricultural production) was selected. Then four villages with a middle socioeconomic level were selected respectively in Fuyu and Dongning counties, and all residents of selected four villages were invited to participate. The residents were informed of the survey about their willingness to undergo free HIV testing at their nearest health care facility via broadcast media and newspapers. After providing informed consent, trained interviewers went door-to-door to invite people and participants complete an anonymous questionnaire (Additional file 1) in a separate room at home. The interviewers provided assistance to some individuals with limited literacy by reading the questionnaire. It took about 15 minutes for participants to complete the questionnaire. A total of 4050 rural and urban residents were approached.,Forty-eight declined to participate. Finally, 4002 were recruited and complete the survey, and the response rate was 98.8%. The Committee on Human Research of Harbin Medical University approved this study.

### Measures

In addition to demographic information, the questionnaire consisted of four other sections: knowledge of HIV/AIDS (fifteen items) [13,14], public attitudes regarding HIV/AIDS (three items) [14], willingness to participate in a free HIV test (one item), and reasons for not being willing to take a free HIV test (one item).

### HIV-related knowledge

The fifteen HIV-related knowledge questions included five items about HIV/AIDS transmission modes, six items about common HIV/AIDS transmission misconceptions and the other four items associated with willingness to participate in free HIV tests in previous studies [15], respectively are AIDS being a contagious disease, an apparently healthy person may being a carrier for HIV, not having a vaccine to protect against HIV, AIDS not being curable, with “do not know” responses scored as incorrect [15,16]. The total score for HIV knowledge, knowledge of HIV transmission modes and HIV transmission misconceptions was the total number of correct responses.

### Public stigma towards people living with HIV/AIDS (PLWHA)

Public stigma, as described by Corrigan and Watson, is the stigmatizing attitudes or reactions of the general population regarding persons with HIV and their family members [17]. Three items were adopted from previous research to reflect public stigma [14]. These items included whether participants were willing to work with PLWHA, accept family members with HIV/AIDS, or have their children study with PLWHA. The stigma towards PLWHA was scored according to the number of responses indicating stigma.

### Willingness to participate in free HIV testing

Willingness to participate in free HIV testing was measured using responses to the question: “If you were offered a free HIV test, with complete secrecy, would you wish to accept it?” In this analysis, “no” and “do not know” responses were combined.

### Statistics analysis

Data were input using Epidata 3.02. Statistical analysis was performed using SAS Software 9.1. Differences among urban residents and rural residents were evaluated by the Student *t*-test or Chi-square test. Univariate log binomial regressions were performed to assess the relationship between each variable and willingness to participate in free HIV tests. Variables in univariate analysis with a significance level < 0.10 were included in the multivariate analysis. Multivariate log binomial regressions adjusted for basic demographic characteristics (i.e., age, gender) were performed to examine the association of HIV-related knowledge and willingness to participate in free HIV tests. *P* < 0.05 was considered statistically significant. We analysed the data separately for urban respondents and rural respondents, because there are large cultural and economic differences between urban areas and rural areas in China.

## Results

The 2018 urban respondents (mean age ± standard deviation (SD): 39.1 ± 13.4 years) consisted of 47.1% male and 52.9% female. The 1984 rural respondents (mean age: 39.6 ±13.5 years) consisted of 49.4% male and 50.6% female. The majority of urban respondents and rural respondents were currently married or cohabitation (74.2%, 83.4%), Han ethnicity (82.6%, 78.7%), lower than 2000 RMB Yuan household per-capita monthly income (88.7%, 89.4%), and had lived in the city for more than two years (83.9%, 92.6%). There were large differences in education levels between urban respondents and rural respondents (P < 0.001). The vast majority of the urban respondents had completed middle school (90.6%), whereas just over half of the rural respondents had done so (53.9%). Demographic characteristics are listed in Table 1.

**Table 1:** Demographic profile of the study subjects

**Characteristic**	**Urban residents**		**Rural residents**		***P*value**
	**Number**	**%**	**Number**	**%**	
**Gender**					0.150
Male	936	47.1	950	49.4	
Female	1053	52.9	973	50.6	
**Ethnicity**					0.002
Han	1647	82.6	1516	78.7	
Minorities	346	17.4	413	21.3	
**Age (years)**					0.134
15–20	138	6.9	148	7.8	
21–50	1372	68.5	1241	65.6	
51–69	492	24.6	504	26.6	
**Marital status**					<0.001
Single	415	21.1	284	14.7	
Married or cohabitating	1461	74.2	1612	83.4	
Divorced or widowed	92	4.7	36	1.9	
**Education level**					<0.001
Primary school and lower	187	9.4	892	46.1	
Middle School	571	28.8	882	45.5	
High school and more	1226	61.8	163	8.4	
**Employment**					<0.001
Unemployed	186	9.4	268	13.8	
Employed	1785	90.6	1668	86.2	
**Length of time lived in city**					<0.001
< 2 years	317	16.1	142	7.4	
≥ 2 years	1654	83.9	1777	92.6	
**Household per capita income (CYN/month)**					0.756
Low income level (<1000)	1283	65.6	1266	66.8	
Medium income level (1000–2000)	451	23.1	429	22.6	
High income level (≥2000)	221	11.3	201	10.6	

Primary school and lower: illiterate or semi-illiterate and elementary school;

Middle school: middle school;

High school and more: high school, technical school, 3-Year college, undergraduate and above.

### HIV-related knowledge

Twenty-eight (0.7%) subjects, who were rural residents, had not heard of HIV/AIDS. The majority of urban residents (82.4%) and rural residents (81.1%) were aware that HIV infection was a contagious disease. For urban residents, the mean scores for the total HIV-related knowledge, HIV/AIDS transmission modes and HIV/AIDS transmission misconceptions respectively was 10.8 (SD = 3.5), 3.8 (SD = 1.4) and 3.8 (SD = 1.9). However, for rural residents, the scores for the total HIV-related knowledge (8.9; SD = 3.8), HIV/AIDS transmission modes (3.5; SD = 1.6), transmission misconceptions (3.1; SD = 2.0) and awareness of respective HIV-related knowledge were all significantly lower than those among urban participants (all *P* <0.05), except for the knowledge that AIDS is a contagious disease. Awareness of five items about HIV/AIDS transmission modes were ranged from 56.6% to 86.8% among all participants, while knowledge of all six items about common HIV/AIDS transmission misconceptions ranged from 21.7% to 76.2%. Details are shown in Table 2.

**Table 2:** HIV/AIDS-related knowledge, Stigma towards people with HIV and willingness to participate in free HIV testing in the study subjects

**Knowledge items**	**Urban residents**		**Rural residents**		***P*value**
	**Number**	**%**	**Number**	**%**	
**Total knowledge score(mean, SD )**	10.8	(3.5)	8.9	(3.8)	<0.001
Is AIDS a contagious disease? (yes)	1644	82.4	1599	81.1	0.285
Can an apparently healthy person be a carrier for HIV? (yes)	1479	74.1	1174	59.6	<0.001
At present, is there a vaccine to protect against HIV? (no)	1085	54.3	753	38.2	<0.001
At present, is AIDS curable? (no)	1393	70.1	996	50.6	<0.001
**Mode of transmission score(mean, SD )**	3.8	(1.4)	3.5	(1.6)	<0.001
1. Can having only one uninfected sex partner reduce the risk of HIV transmission? (yes)	1221	61.4	1112	56.6	0.001
2. Can use of condoms reduce the risk of transmission of HIV? (yes)	1424	71.3	1264	64.3	<0.001
3. Can sharing contaminated needles and syringes cause AIDS?	1609	80.6	1522	77.3	0.011
4. Can receiving blood and blood products lead to AIDS? (yes)	1735	86.8	1583	80.3	<0.001
5. Can a pregnant woman transmit HIV to her baby? (yes)	1624	81.4	1406	71.4	<0.001
**Misconceptions of transmission score(mean, SD )**	3.8	(1.9)	3.1	(2.0)	<0.001
1. Can mosquito bites transmit HIV? (no)	739	37.0	427	21.7	<0.001
2. Can eating food together transmit HIV? (no)	1390	69.6	1084	55.1	<0.001
3. Can coughing or sneezing lead to AIDS? (no)	1353	67.8	1060	53.8	<0.001
4. Can shaking hands, hugging, or kissing cause AIDS? (no)	1470	73.6	1276	64.7	<0.001
5. Can sharing the same office cause AIDS? (no)	1523	76.2	1236	62.7	<0.001
6. Can using the same toilet, shower, or swimming pool transmit HIV? (no)	1097	54.9	976	49.5	<0.001
**Stigma score(mean, SD )**	0.6	(0.9)	0.8	(0.9)	<0.001
1. Is willing to work together with PLWHA?	519	26.3	650	33.8	<0.001
2. Is willing to accept family members with HIV/AIDS?	189	9.6	192	10.0	0.706
3. Is willing to have their children study together with PLWHA?	454	23.0	648	33.8	<0.001
**willingness to participate in free HIV testing**	1438	73.0	1516	78.8	<0.001

### Public stigma towards PLWHA

Overall, 26.3% of urban respondents and 33.8% of rural respondents thought that PLWHA should be kept away from their colleagues (*P* < 0.001); 9.6% and 10.0%, respectively, were unwilling to accept family members with HIV/AIDS (*P* = 0.706); and 23.0% and 33.8%, respectively, would not allow their children to study with PLWHA (*P* < 0.001). Mean score on stigma towards PLWHA among urban participants was 0.8 (SD = 0.9), significantly higher than that among rural participants (0.6; SD = 0.9) (*P* < 0.001).

### Willingness to participate in free HIV tests

The proportions of participants who were willing to participate in free HIV testing among urban participants (73.0%) was significantly lower than that among rural participants (78.8%; *P* < 0.001). Univariate and multivariate log binomial regression analyses to identify factors associated with willingness to accept free HIV testing are shown in Table 3.

**Table 3:** Univariate and multivariate analysis of factors associated with willingness to participate in free HIV testing

**Variable**		**Urban residents**		**Rural residents**				
		**Crude RR**	**Adjusted RR**	**Crude RR**	**Adjusted RR**			
**Gender**	Male	1.00	1.00	1.00	1.00
	Female	0.95(0.90–1.00)	0.99(0.94–1.05)	0.93(0.89–0.98) **	0.94(0.90–0.98) **
**Ethnicity**	Han	1.00		1.00	1.00
	Minorities	0.99(0.92–1.06)		0.94 (0.88–1.00) *	0.97 (0.92–1.03)
**Age (Years)**	15–20	1.00	1.00	1.00	1.00
	21–50	1.10(0.97–1.24)	1.13(0.99–1.28)	1.24(1.10–1.40) **	1.14 (0.99–1.32)
	51–69	1.04(0.91–1.18)	1.09(0.95–1.27)	1.21(1.06–1.37) **	1.13 (0.98–1.32)
**Marital status**	Single	1.00	1.00	1.00	1.00
	Married/cohabitating	0.95(0.89–1.01)	0.94(0.88–1.01)	1.10(1.02–1.18) *	1.05(0.97–1.15)
	Divorced/widowed	0.87(0.74–1.02)	0.84(0.71–0.99) *	1.17 (1.00–1.37) *	1.07(0.91–1.27)
**Education**	Primary school or less	1.00		1.00	
	Middle school	0.94 (0.85–1.04)	0.89(0.81-0.89)*	1.02 (0.97–1.07)	0.99(0.95–1.04)
	Over high school	0.99 (0.91–1.09)	0.90(0.83-0.99)*	1.05 (0.97–1.14)	0.98(0.90–1.05)
**Employment**	Unemployed	1.00		1.00	
	Employed	1.06 (0.96–1.17)		0.94(0.88–1.00)	
**Time in city**	< 2 years	1.00		1.00	
	≥ 2 years	0.95 (0.89–1.02)		0.95 (0.88–1.03)	
**Household per-capita income (CNY/month)**	<1000	1.00	1.00	1.00	1.00
	1000–2000	1.09(1.02–1.16) **	1.07(1.01–1.14)*	1.00 (0.95–1.06)	0.98 (0.93–1.03)
	≥2000	1.03 (0.94–1.12)	1.01(0.92–1.10)	0.93 (0.85–1.01)	0.92 (0.85–0.99) *
**HIV/AIDS Knowledge**					
Is AIDS a contagious disease?		1.10(1.02–1.19) *	0.99(0.92–1.08)	1.11 (1.03–1.19) **	1.02(0.95–1.10)
Can an apparently healthy person be a carrier for HIV?		1.14(1.06–1.22) **	1.12(1.03–1.21)**	1.13 (1.08–1.19) **	1.07(1.01–1.13) *
At present, is there a vaccine to protect against HIV?		1.05 (0.99–1.10)	1.00(0.94–1.06)	1.10 (1.05–1.15) **	0.99(0.94–1.04)
At present, is AIDS curable?		1.02 (0.96–1.08)	0.95(0.89–1.02)	1.11 (1.06–1.17) **	1.02 (0.97–1.08)
**Awareness of transmission modes score**		1.05(1.03–1.07) **	1.02(1.00–1.05)	1.05(1.03–1.06)**	1.03(1.01–1.06)**
**Awareness of transmission misconceptions score**		1.03(1.02–1.05) **	1.02(1.00–1.04) *	1.03 (1.02–1.04) **	1.01(0.99–1.02)
**Stigma attitude score**		1.00(0.97–1.03)		1.02(1–1.05)	
**Total Knowledge score**		1.02(1.01–1.03) **		1.02(1.01–1.03) **	

RR: Relative risk (95% CI); * P <0.05; **P <0.01.

Crude RR: Relative risk of univariate analysis.

Adjusted RR: Basic demographic characteristics (i.e., age, gender) and variables that were associated with willingness to accept free HIV testing in univariate analysis among urban residents at the 0.10 significance level were put stepwise in a multivariate log binomial regression model.

The univariate analysis showed that greater total knowledge about HIV/AIDS was significantly associated with greater willingness among urban residents (RR = 1.02, 95%CI: 1.01–1.03, *P* < 0.001) and among rural residents (RR = 1.02, 95%CI: 1.01–1.03, *P* < 0.001). Stigma was not associated with willingness among urban participants (RR = 1.00, 95%CI: 0.97–1.03, P = 1.000), while there was a trend for an association with willingness among rural participants (RR = 1.02, 95%CI: 1.01–1.03, *P* < 0.067).

Results of multivariate regression analysis among urban participants adjusted for age, gender, education, marital status, and income showed that a medium income level (RR = 1.07, 95%CI: 1.01–1.14, *P* = 0.021), having a greater knowledge of HIV transmission misconceptions (RR = 1.02, 95%CI: 1.00–1.04, *P* = 0.021) and being aware that an apparently healthy person can be a carrier for HIV (RR = 1.12, 95%CI: 1.03–1.21, *P* = 0.007) was significantly associated with greater willingness to accept a free HIV test (Table 3). However, a higher education level (middle school vs. primary school or less: RR = 0.89, 95%CI: 0.81–0.98, *P* = 0.017; high school vs. primary school or less: RR = 0.90, 95%CI: 0.83–0.99 *P* = 0.032), and being divorced or widowed (RR = 0.84, 95%CI: 0.71–0.99, *P* = 0.042) were significantly associated with a decreased willingness to accept a free HIV test (Table 3). Multivariate regression analysis among rural participants adjusted for age, gender, education, ethnicity, marital status, and income showed that having a greater knowledge of HIV transmission modes (RR = 1.03, 95%CI: 1.01–1.06, *P* = 0.013), and being aware that an apparently healthy person could be a carrier for HIV (RR = 1.07; 95%CI: 1.01–1.13 *P* = 0.019) were significantly associated with greater willingness to participate in HIV testing. However, being female (RR = 0.94, 95%CI:16 0.90–0.98 P = 0.006) and high income level (RR = 0.92, 95%CI: 0.85–0.99 P = 0.034) were significantly associated with lower willingness to participate in HIV testing.

The main reasons for not being willing to accept free HIV tests among urban participants included believing that they were unlikely to have been exposed to HIV (38.7%), not having enough time (19.7%), fear of stigma (14.7%), and not wanting to know the result (11.1%). Reasons were similar among rural participants: 58.3%, 17.6%, 17.0%, and 15.0%, respectively. Other reasons given were “do not trust the result to be confidential,” “old age” (i.e., believed oneself too old to be important to health), and “worry about infection in the course of testing.”

## Discussion

The results of our study showed that the willingness of urban residents (73.0%) to undergo HIV testing was significantly lower than that among rural residents (78.8%). Willingness to participate in free HIV testing were slightly lower than those found in studies carried out among high-risk populations in China (from 78.0% to 94.0%) [12,18-21]. Willingness to undergo HIV testing in this study (78.8% rural; 73.0% urban) was greater than that of participants in the only published study of the general public, aged 18–45 years, carried out between October 2005 and February 2006 in Guizhou Province, China (43.5%) [22]. The difference may be due to time trends. With the further promotion of HIV knowledge and relevant policy, people may be more willing to accept a free HIV test. Simultaneously, the difference may also be due to the possibility of undergoing HIV testing in the nearest health care facility, which offers greater accessibility and convenience, compared with accepting a free HIV test in a VCT centre in the study in Guizhou Province. There are relatively few VCT centres in China, especially in rural areas, and they are often at a distance from the area of residence, thus visiting a VCT centre incurs costs in terms of travel and time. A study of Chinese sex workers found that participants were more willing to be tested near their workplaces [23]. In September 2006, to reduce the number of persons with undiagnosed HIV infection, the United States Centers for Disease Control issued recommendations to implement HIV screening as part of routine medical care for all persons aged 13–64 years in all health care settings, based on ensured privacy and confidentiality and referral to prevention and clinical services as necessary [24]. However, further studies are needed to find suitable protocols and sites of HIV testing among high-risk groups and the general population, such as combining HIV testing with other health examinations, and integrating VCT services into existing health centres in China.

HIV-related knowledge awareness was lower than that found in studies among high-risk populations in Heilongjiang Province [25,26]. In our study, overall HIV-related knowledge awareness among rural residents was lower than that among urban residents. Similar results were found in studies in 2005 in Hubei Province and Liaoning Province, China [27,28]. This indicates a need for further education about HIV in rural areas in China. Meanwhile, While 45.0% of urban respondents and 38.0% of rural respondents could correctly identify all five items regarding important modes of HIV/AIDS transmission, only 19.8% of urban respondents and 9.3% of rural respondents could identify all six common HIV/AIDS transmission misconceptions (*P* < 0.001). Similar results were found in the study in southwest China in 2003 [29] and in Xinjiang in 2006 [30]. Low awareness about HIV/AIDS transmission misconceptions highlights the need for further education about these misconceptions.

Univariate analysis among both urban and rural residents showed that greater total HIV/AIDS knowledge was significantly associated with greater willingness to participate in free HIV testing, which suggests that the wider and more accurate an individual’s HIV-related knowledge, the more willing he or she will be to accept a free HIV test. In our study, stigma was not associated with willingness to undergo testing in urban areas, while there was a trend toward an association in rural areas. Liu’s path analysis in a rural area of China found that stigmatizing attitudes were only indirectly associated with intention to disclose HIV serostatus through perceived stigma [31]. Further study is needed to document the relationship between willingness to participate in free HIV tests and other stigma indicators.

Multivariate regression analysis of willingness to participate in HIV testing among both urban and rural residents showed that the awareness that an apparently healthy person can be a carrier of HIV was significantly associated with greater willingness to participate in HIV testing. This finding is consistent with Sarker’s study in Burkina Faso [32] and Gage’s study in Uganda [33]. There was no significant difference between male and female among urban residents. However, males were more willing to participate in free tests than females among rural residents, similar to results found in the study by Liu among rural residents in China [20]. It is possible that rural females have less access to health information in China. Urban residents with lower education level were more willing to participate in free HIV testing. This result is consistent with the findings among the general population in the United States [34]. Urban residents with better knowledge of HIV transmission misconceptions were more willing to participate in free HIV testing. Possible explanations are that misconceptions about modes of HIV transmission could heighten the fear of infecting another person or being infected via daily life contact, thus reducing willingness to participate HIV testing. In contrast, rural residents who had better knowledge of HIV transmission modes were more willing to participate in free HIV testing. It may be that knowledge of HIV transmission modes strengthens the perception of infection risk, thus increasing willingness to participate in HIV testing. We conclude that HIV/AIDS education needs to be improved to increase willingness to undergo HIV testing among the general population in Heilongjiang, with particular emphasis on HIV transmission modes in rural areas, and on dispelling misconceptions about HIV transmission modes in urban areas.

The most common reason for unwillingness to accept free HIV tests was participants’ belief that they had not personally been exposed to HIV. The results suggest that when people are conscious of their risk for HIV, they are more willing to participate in HIV testing. Another reason was that respondents were not willing to know their test results. This suggests that the respondents thought they could not benefit from awareness of their serostatus. Therefore, programs to improve knowledge about HIV treatment should be carried out, so that people know that early and compliant treatment can improve the quality and length of HIV carriers’ and AIDS patients’ lives, and mother-to-child transmission of HIV can be prevented [35-38]. Efforts should also be promoted to inform people about Chinese HIV-related policies, such as the “Four Free and One Care” policy [39]. The policy includes free antiretroviral drugs for those who cannot afford to pay, testing, prevention of mother-to-child transmission, and schooling of orphans [40].

The study has some limitations. First, although the study results indicates willingness to test in the general population are high, not all who are willing to test for HIV will actually do so over time. Therefore, further studies as to the circumstances under which residents will actually accept testing are needed (e.g., education about HIV-related knowledge and policies, suitable styles and sites of HIV testing, privacy and confidentiality protection, and clinical services). Secondly, our questionnaire might not reflect all aspects of stigma or bias, as we only studied the association of public stigmatising attitudes and willingness to participate in HIV testing. Thirdly, as the sample was not a perfect random sample, some selection bias may exist. another limitation of the analysis are non-response bias, however the relatively high response rate (98.8%) might minimise this bias. Finally, owing to the cross sectional nature of this study, these data should be interpreted as associations rather than implying causality. Despite the limitations, our findings provide valuable information for HIV test. The findings highlight that education on HIV/AIDS needs to be improved to increase willingness for HIV testing among the general public, especially emphasising knowledge of HIV transmission modes in rural areas and countering misconceptions of HIV transmission modes in urban areas.

## Conclusions

Our results indicate the willingness of general residents in Heilongjiang, Northeast China, to participate in free HIV testing in the nearest health care setting is high, and is higher in rural residents than in urban residents. However, further studies are needed to find suitable protocols and sites of HIV testing among high-risk groups and the general population. Our results indicate that knowledge of HIV transmission misconceptions are associated with willingness to accept free HIV testing among urban residents, while knowledge of HIV transmission modes are associated with willingness to accept free HIV testing among rural residents. Therefore, we believe that HIV/AIDS education needs to be improved to increase willingness to undergo HIV testing among the general population in Heilongjiang, with particular emphasis on HIV transmission modes in rural areas, and on dispelling misconceptions about HIV transmission modes in urban areas.

## Competing interests

We declare no competing interests.

## Authors’ contributions

Yuan Lili was responsible for data acquisition, analysis, interpretation, drafting and production of the final manuscript. Li Xin was responsible for study design, data acquisition, analysis, interpretation. Shi Jingli was responsible for study design, date acquisition. Jiang Liying was responsible for date acquisition, analysis, interpretation. Zhang Chundi was responsible for study design, acquisition of date. Yang Xiujing was responsible for study design, acquisition of date. Zhao Yashuang was responsible for study design, analysis, interpretation, drafting and production of the final manuscript. All authors read and approved the final manuscript.

## Funding

This study was supported by Health Department of Heilongjiang Province, China (2006–308).

## Pre-publication history

The pre-publication history for this paper can be accessed here:

http://www.biomedcentral.com/1471-2334/12/256/prepub

## Supplementary Material

Additional file 1 Health Survey Questionnaire. Click here for file
