# Supplementary material for: Factors associated with willingness to participate in free HIV test among general residents in Heilongjiang, Northeast China
Source: BMC Infect Dis. 2012 Oct 12;12:256. doi: 10.1186/1471-2334-12-256 (PMC3482579; doi:10.1186/1471-2334-12-256)
Supplement: Additional file 1 — Health Survey Questionnaire. [file 1471-2334-12-256-S1.doc]

Number: □□□□□□

**Health Survey Questionnaire**

Survey Date: (Year), (Month),

Hello, we are conducting a survey with the goal of understanding your knowledge and attitudes towards several health problems. Please don’t worry，this survey is anonymous。We hope that your answers will be your own personal views and beliefs. This survey will take around 15 minutes; please carefully read the questionnaire and then fill your answers into the parentheses; when filling in the blanks answer according to your actual situation. We hope you will support us in this work. Thank you very much!

1. **Basic Situation**

1. （ ）Gender： ① Male ② Female

2. （ ）Birth Date: (Year), (Month)

3. （ ）Ethnicity： ① Han ② Manchu ③ Mongol ④ Chaoxian Ethnicity ⑤ Korean Ethnic Minority ⑥ Other ______

4. （ ）Marital Status： ① Single ② Married ③ Cohabitation ④ Divorced or Spouse Died

5. （ ）Education Level： ① Illiterate or Semi-Illiterate ② Elementary School ③ Middle School ④ High School/Technical School ⑤ 3-Year College ⑥ Undergraduate and Above

6. （ ）Profession： ① Student ② Teacher ③ Child-Care Worker or Housekeeper ④ Food Services ⑤ Business Person

⑥ Worker ⑦ Medical Personnel ⑧ Civilian/Peasant Worker ⑨ Peasant

⑩ Herder Fisherman/Woman Cadre Worker Retiree

Household Duties/Awaiting Employment Other:___________

7.（ ）Length of Time Lived in this City： ① ＜3 Months ② 3～6Months ③ 6～12 Months ④ 1～2 years ⑤ More than 2 years

8. （ ）Household Per-Capita Income： ① Under 500 yuan/month ② 500～1000 yuan/month ③ 1000～2000 yuan/month ④ 2000～3000 yuan/month ⑤ 3000～4000 yuan/month ⑥ More than 4000 yuan/month

B. **Knowledge, Understanding, and Attitudes**

1. （ ）How much do you understand AIDS?

① A lot ② Somewhat ③A little ④ Not at all

1. （ ）Are people infected with HIV and people with AIDS the same thing? ① Yes ② No ③ Don’t Know
2. (）Is AIDS contagious? ① Yes ② No ③ Don’t Know
3. （ ）Can an apparently healthy person be a carrier for HIV?

① Yes ② No ③ Don’t Know

1. （ ）Can receiving blood fluids or blood products with HIV lead to transmission of HIV?

① Yes ② No ③ Don’t Know

1. （ ）Can sharing acupuncture equipment and needles with an HIV infected person result in transmission of HIV? ① Yes ② No ③ Don’t Know
2. （ ）Can the proper use of a condom with all sexual activity reduce the risk of transmission of HIV?

① Yes ② No ③ Don’t Know

1. （ ）Can maintaining a monogamous sexual relationship with a partner who is not infected with HIV reduce the risk of transmission of the virus? ① Yes ② No ③ Don’t Know
2. （ ）Can a pregnant women who is infected with HIV pass the virus on to her child? ① Yes ② No ③ Don’t Know
3. （ ）Can eating food together with HIV-infected people or people

with AIDS transmit HIV? ① Yes ② No ③ Don’t Know

1. （ ）Can insect bites transmit HIV? ① Yes ② No ③ Don’t Know
2. （ ）Can coughing or sneezing transmit HIV?① Yes ② No ③ Don’t Know
3. （ ）Can shaking hands, hugging, or kissing transmit HIV?

① Yes ② No ③ Don’t Know

1. （ ）Can sharing the same office with someone with AIDS and using the same appliances transmit HIV? ① Yes ② No ③ Don’t Know
2. （ ）Will using the same toilet, shower, or swimming pool as an HIV infected person transmit HIV? ① Yes ② No ③ Don’t Know
3. （ ）Will using unsterilized hair styling/cosmetology appliances transmit HIV? ① Yes ② No ③ Don’t Know
4. （ ）At present, is there a vaccine to protect against HIV?

① Yes ② No ③ Don’t Know

1. （ ）At present, infection with HIV curable?

① Yes ② No ③ Don’t Know

1. Which of the following things do you think will reduce the risk of infection with HIV? (You can choose several, write the numbers in the parentheses)（ ）

① Limit one’s sexual behavior，not have casual sex、do not engage in prostitution，reduce number of sexual partners； ② Do not use illegal drugs； ③ Not injecting blood products that have been officially tested ④ When using needles、 syringes、 dentistry tools or these types of instruments，they must be disposable or have undergone strict sterilization； ⑤ Not using unsterilized instruments to get one’s ears pierced、 get tattoos、or at the beauty parlor； Not sharing razors or toothbrushes with others； ⑥ If pregnant, be prepared to undergo HIV antibody screening and counseling; ⑦ Properly use a condom； ⑧ Treat other illnesses promptly。

1. （ ）Would you like to know more about HIV?

① Yes ② No ③ Don’t Know

If so, in what ways would you like to receive information about HIV?

（You can choose several）（ ）

① Presentation of information by a schoolmate ② Specialized Publicity ③ Book、Newspaper ④ Television、Broadcast ⑤ Classroom Setting ⑥ Medical Department ⑦ Parents ⑧ Friend ⑨ Internet

1. （ ）If in your class at school a classmate was infected with HIV, you would:

① Suggest the work unit expel him/her； ② Suggest that he/she quit or resign； ③ agree to him/her staying in the work unit, you wouldn’t worry； ④ Not know what to do； ⑤ Other (Please describe）____________

1. （ ）If in your family someone was infected with HIV, you would: ① Make him/her leave home to go live somewhere else； ② Allow him/her to keep living at home, but eat meals separately； ③ Allow him/her to stay at home, and take care of him/her； ④ Not know what to do； ⑤ Other（Please describe）____________
2. （ ）If among your child’s classmates there was someone infected with HIV, you would:

① Suggest that the school expel the classmate or make him/her attend isolated class； ② Suggest that the school show the classmate concern and care； ③ Make your child switch schools； ④ Not know what to do； ⑤ Other（Please describe）____________

1. （ ）Do you approve of HIV infected persons’ or HIV infected-persons’ right to privacy

① Yes ② No ③ Don’t Know

1. （ ）Do you think that people who are infected with HIV have the infection as a result of bad lifestyles? ① Yes ② No ③ Don’t Know
2. （ ）What do you think your risk of becoming infected with HIV is? ① High ② Medium ③ Low ④ Not possible ⑤ Don’t know
3. （ ）What do you think the most important measures to take to prevent HIV transmission?（You can choose several） ① Propaganda and education ② Prohibiting prostitution、Illegal drug use ③ Reducing the number of sex partners， Advocating condom use ④ Rigorously control blood products ⑤ Don’t know

C. **Attitudes Toward Free Testing**

1.（ ）If you were offered a free HIV test, with complete secrecy, would you wish to accept testing? ① Yes ② No ③ Don’t Know

2.（ ）If you were offered a free HIV test together with a Hepatitis B test, with complete secrecy, would you wish to accept testing? ① Yes ② No ③ Don’t Know

3. If you were not willing to accept the free HIV test, what were your reasons?（You can pick several）（ ）

① Fear of stigma ② Not enough time ③ Don’t want to know the result ④ Don’t think oneself could become infected with this disease ⑤ Other（Please describe）_____

4. If you would accept HIV testing how would you like to be notified of the results? （You can pick several）：（ ）

① Face-to-face discussion with an assigned person ② Written notice ③ Telephone notification ④ Personally call to inquire ⑤ Personally go to testing location to enquire ⑥ Don’t want to know the result，prefer it to remain a secret ⑦ Other（Please describe ）_________

**End of questionnaire, Thank you for your help.**

Survey Taker’s Name：_________
